# Determinants of malaria treatment delay in northwestern zone of Tigray region, Northern Ethiopia, 2018

**DOI:** 10.1186/s12936-019-2992-7

**Published:** 2019-11-09

**Authors:** Afewerki Tesfahunegn, Dawit Zenebe, Alefech Addisu

**Affiliations:** 1 Epidemiology Department, School of Public Health, Mekelle University, Mekelle, Ethiopia; 2 Federal Ministry of Health, Mekelle, Ethiopia

**Keywords:** Malaria, Malaria treatment, Malaria treatment delay, Health-seeking for malaria treatment, Northwestern zone of Tigray

## Abstract

**Background:**

Globally malaria affects 212 million people and causes 438,000 deaths each year. Ensuring early and timely treatment of malaria is important for preventing and controlling of life-threatening complications and further transmission. Even though malaria treatment is widely available in Ethiopia, 47–84% of patients present after 24 h of onset of first symptoms. This study assessed the determinants of delay for malaria treatment in Tigray, Ethiopia.

**Methods:**

A health facility-based case–control study design in northwestern zone of Tigray was conducted from September 2018 to January 2019. All the study participants enrolled were confirmed malaria patients (by microscopy or rapid diagnostic test) and who sought treatment. Cases were defined as malaria patients who sought treatment after 24 h of the onset of the first symptom and control were those who sought treatment within 24 h onset of symptom. A structured questionnaire was used to collect data on the determinants of malaria treatment delay. Data were entered into EpiInfo 7.0 and exported to SPSS 20.0 for analysis. Binary logistic regression was computed to identify predictors of delay for malaria treatment.

**Results:**

In total 161 cases and 161 controls were identified. Being residents of Tahtay Adyabo district (AOR = 2.84, 95% CI 1.29–6.27), having no formal education (AOR = 2.39, 95% CI 1.09–5.22), the decisions to seek health care being taken by the patient (AOR = 2.38 95% CI 1.09–5.2), the decisions to seek health care being taken by their fathers (AOR = 2.52, 95% CI 1.13–5.62), and having good knowledge about malaria symptoms (AOR = 2.02, 95% CI 1.21–3.39) were found determinants of delay for malaria treatment.

**Conclusion:**

In this study, delays in obtaining treatment for malaria were associated with having no formal education, knowing about the signs and symptoms of malaria, living in Tahtay Adyabo district, and decision-making on seeking malaria treatment. The results suggests having treatment commenced at sites closer to the community and strengthened awareness-raising activity about the importance of early seeking for all with malaria-like symptoms, especially for household heads would contribute to improved treatment and reduced complications from malaria.

## Background

Malaria is caused by parasites of *Plasmodium* species and transmitted by female *Anopheles* mosquitoes. Malaria affects 212 million people and causes 438,000 deaths worldwide annually with 90% from Africa of which 76% from Sub Saharan Africa [1, 2]. In Ethiopia, about 52 million (68%) people live in malaria risk areas; 2.2 million people are affected, including 14% from Tigray region [3, 4].

In Ethiopia, effective and affordable malaria diagnosis and treatment services are comprehensively available in more than 90% of health centres and hospitals and in 77% of health posts. The health institutions use microscopy or rapid diagnostic tests (RDTs) as diagnostic methods. Treatment of malaria is an essential component of anti-malarial interventions in the country in addition to malaria prevention activities. Uncomplicated malaria can progress rapidly to complicated malaria and cause death if not treated on time. Therefore, early diagnosis and promote effective treatment within 24 h of the onset of malaria is important to prevent and control the development of life-threatening complications and further transmission. Ethiopia has set a malaria elimination goal by the year 2020. Early treatment of cases is among the vital component of malaria control and elimination strategies [3–10].

The proportion of people who delayed seeking malaria diagnosis and treatment is relatively high in the districts of the northwestern zone of Tigray region and the whole of Ethiopia. Studies conducted around the country, including in Tigray, showed that delay to treatment among malaria patients is common. In these studies, 47% to 84% of patients with malaria were presented for treatment after 24 h of symptom onset [11–17]. These studies conducted around the world assessed that sociodemographic factors (age, residency, and monthly income), behavioural factors, and physical factors were found predictive factors for delay in malaria treatment among under-five children [13, 15, 18–27].

Although there is some literature that assessed the determinants of delay in malaria treatment in children, this crucial health problem has not been well studied in the general community of the Tigray region of Ethiopia. This region is one of the malaria-endemic areas and has a high level of delay in malaria treatment. In order to achieve the 2020 malaria elimination goal increasing early treatment is essential. The results of this study could be helpful for the planning of malaria elimination strategies. This study assessed the determinants of delay for malaria treatment in northwestern zone of Tigray, Ethiopia.

## Methods

### Study area and period

This study was conducted in the northwestern zone of Tigray region, Northern Ethiopia from September 2018 to January 2019. The northwestern zone is one of the seven administrative zones of Tigray. According to the central statistical agency, the population size of the zone is approximately one million of which 50% are females. The zone had one general hospital and 41 health centres. The zone has both lowland and semi highland conditions with an average temperature of 31 °C and one cycle of rainfall per year. All the districts located in this zone are malaria-endemic areas.

### Study design

Health facility-based unmatched case–control study design was conducted with one to one case to control ratio to assess determinants of malaria treatment delay in health facilities.

### Study population

Malaria patients who sought treatment in the health facilities of the northwestern zone of Tigray from September 2018 to January 2019 and were confirmed for *Plasmodium* species by blood film examination or rapid diagnostic test (RDT) were enrolled in the study.

### Operational definition

Case was identified as a patient who sought treatment after 24 h of the onset of the first symptoms from September 2018 to January 2019 [6]. Control was identified as a patient who sought treatment within 24 h of the onset of the first symptoms from September 2018 to January 2019 [6].

On the questionnaire, ‘Delayed for malaria treatment’ was defined as a patient who came to the health facility for malaria treatment and confirmed by microscopy or RDT for *Plasmodium* species and presents after 24 h of the onset of the first symptom. ‘Timely treated’ was defined as a patient who came to the health facility for malaria treatment within 24 h of the onset of the first symptom. Good knowledge of malaria was defined as a participant scoring above the mean of malaria knowledge questions Poor knowledge was defined as a participant scoring below the mean.

### Sample size determination

The sample size was determined by double population proportion formula for unmatched case–control study in EpiInfo 7.0 stat calc. The calculation was computed by taking the assumption of: “fear of side effect” with odds ratio of 4.96, percent of control exposed 2.6%, percent of cases exposed 11.7%, with 95% confidence interval, by consideration of 80% power, case to control ratio of 1:1, and by adding 10% non-response rate [20]. Malaria patients were recruited on a one to one case to control ratio: 322 study participants with 161 cases and 161 controls.

### Sampling technique and procedure

A two-stage sampling technique was applied to recruit the participants. First, nine health centres from the total 41 health centres in the zone were selected randomly. The study participants were selected from each health centre by a systematic random sampling technique. A sampling frame was developed by assessing the data from the last 3 years of each health centre. Selection of participants was then allocated proportionally based on the previous year’s patient flow.

### Variables

Dependent variable was timing of malaria treatment defined as (delayed or timely treated). Independent variables are: Socio-demographic variables (age, gender, marital status, religion, ethnic group, residence, educational level, occupation, family size and average monthly income); behavioural factors (knowledge on malaria, history of death from family from any cause, fear of cost, fear of side effect, taking traditional medicine, alcohol drinking, and distrust of health providers); and physical and environmental variables (distance to health facilities, availability of transport access, and type of transportation).

### Data collection tool and technique

A structured and pre-tested questionnaire was used by adapting from relevant literature on this topic. The questionnaire had an introduction, socio-demographic, behavioural factors, and physical and environmental factors. Informed consent was obtained before the interview commenced. The questionnaire was translated into the local language (*Tigrigna*) and back to English by different personnel to maintain consistency of the questions. After the patients were diagnosed by a clinician in the health centre, data were collected by interviewer-administered questionnaire from those patients/caregivers and their respective medical records by nine trained data collectors and three supervisors from the health centres.

### Data quality control

A 2-day intensive training was provided to the nine data collectors and three supervisors for 2 days on how to fill the questionnaire and on methods of sampling and obtains consent from participants. The questionnaire was pretested in 32 participants and some amendments were made accordingly. Each day filled questionnaire were checked for completeness and validity before each study participant left the health centres. The collected data were cross-checked by supervisors and principal investigators.

### Data management and analysis

Data were entered into Epi info 7.0 after it was cleaned and coded, then exported to SPSS 20.0 for analysis. Descriptive analyses were accompanied by the frequency with percentage for categorical variables and median and Inter Quartile Range (IQR) for continues variables and normality was also checked by a graph.

Binary logistic regression analyses were performed to identify factors associated with malaria treatment delay. Variables with *P* value < 0.2 in bivariate logistic regression was entered to multivariable logistic regression analysis to identify the predictor variables. Multi-collinearity was checked by variance inflation factor (VIF) and value less than 10 was included in the model. Model fitness was checked by Hosmer and Lemeshow’s goodness-of-fit and also interaction was tested. Bivariate and multivariable analyses were conducted and finally, P-value ≤ 0.05 was taken as a statistically significant factor and data were presented using adjusted odds ratio (AOR) with 95% confidence interval.

### Ethical consideration

Ethical clearance was obtained from the institutional review board of Mekelle University, College of Health Sciences then supportive letter was obtained from Tigray Regional Health Bureau to the study area. Participants/caregivers consented and minors have assented verbally. Confidentiality was maintained by not having personal identifying data on the questionnaire (i.e. anonymous). The data was not used for other purposes than the study objective.

## Results

### Socio-demographic characteristics

**Fig. 1:**
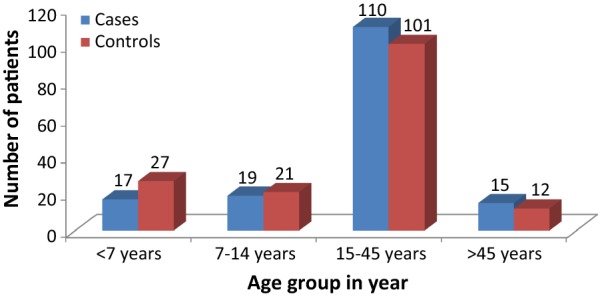
Age category of participants in the northwestern zone of Tigray, Northern Ethiopia, 2018

A total of 322 participants were included in this study with 100% response rate. The median age of cases and controls were 20 (IQR = 15–34) years and 20 (IQR = 13–30) years respectively. Most (68%, 110/161) of the cases and (63%, 101/161) of the controls were in the age group of 15–45 years (Fig. 1). Above two-thirds (72%, 116/161) of the cases and (65%, 105/161) of the controls were males. Seventy (43.5%) of the control and (51.6%, 83/161) of the cases identified farming as their primary occupation. Regarding educational status, (44.1%, 71/161) of control and (52.8%, 85/161) of the case had not attended formal education (Table 1).

**Table 1:** Socio-demographic characteristics of participants in northwestern Tigray region, Northern Ethiopia, 2018

Variable	Category	Cases n = 161 No (%)	Control n = 161 No (%)
Gender	Male	116 (72)	105 (65.2)
	Female	45 (28)	56 (34.8)
Age group	< 7	17 (10.6)	27 (16.8)
	7–14	19 (11.8)	21 (13)
	15–45	110 (68.3)	101 (62.7)
	> 45	15 (9.3)	12 (7.5)
Place of residence	Laelay Adyabo	91 (56.5)	90 (55.9)
	Tahtay Adyabo	42 (26.1)	30 (18.6)
	Asgede Tsimbla	28 (17.4)	41 (25.5)
Occupation	Farmer	83 (51.6)	70 (43.5)
	Merchant	5 (3.1)	5 (3.1)
	Housewife	7 (4.3)	3 (1.9)
	Student	42 (26.1)	48 (29.8)
	Government worker	2 (1.2)	8 (5)
	Daily labourer	5 (3.1)	2 (1.2)
	Preschool age	17 (10.6)	25 (15.5)
Marital status	Married	75 (46.6)	63 (39.1)
	Never married	80 (49.7)	90 (55.9)
	DW	6 (3.7)	8 (5)
Educational status	No formal education	85 (52.8)	71 (44.1)
	Primary	58 (36)	64 (39.8)
	Secondary and above	18 (11.2)	26 (16.1)
Monthly income status	< 500 ETB	39 (24.2)	30 (18.6)
	500–1000 ETB	94 (58.4)	107 (66.5)
	> 1000 ETB	28 (17.4)	24 (14.9)

*DW* divorced and widowed

### Behavioural and physical characteristics of delay in malaria treatment

The majority 84.5% (138/161) of the controls and 88.2% (142/161) of the cases had *Plasmodium falciparum* species and the rest had *Plasmodium vivax*. Concerning medication, 6.4% (10/161) of the cases and 12.4% (20/161) of the controls had taken any medication in their house for the current illness before they came to the health centre; and 19.3% (31/161) of the controls and 17.4% (28/161) of the cases feared side effect of malaria; while 10.6% (17/161) of the cases and 10.1% (16/161) of the controls feared the cost of malaria treatment.

Regarding the decision-making to seek health care, 44.7% (72/161) of controls and 50.9% (82/161) of cases decided to seek health care by the patient themselves. Thirty-four percent (55/161) of controls and 46% (74/161) of cases had good knowledge of malaria symptoms (Table 2).

**Table 2:** Behavioural and physical characteristics of participants in northwestern Tigray, Ethiopia, 2018

Variable	Category	Cases n = 161 No (%)	Control n = 161 No (%)
Knowledge of malaria symptoms	Good	74 (46)	55 (34.2)
	Poor	87 (54)	106 (65.8)
Knowledge of malaria mode of transmission	Good	142 (88.2)	133 (82.6)
	Poor	19 (11.8)	28 (17.4)
Knowledge of malaria at risk population	Good	62 (38.5)	82 (50.9)
	Poor	99 (61.5)	79 (49.1)
Knowledge of malaria prevention	Good	102 (63.4)	111 (68.9)
	Poor	59 (36.6)	50 (31.1)
Who decides to seek health care	Patient	82 (50.9)	72 (44.7)
	Father	63 (39.1)	63 (39.1)
	Mother	16 (9.9)	26 (16.1)
Dead family member in the last month by any cause	Yes	2 (1.2)	6 (3.7)
	No	159 (98.8)	155 (96.3)
Taking care previously in the health centre	Yes	144 (89.4)	144 (89.4)
	No	17 (10.6)	17 (10.6)
Taking care on time previous time	Yes	119 (79.3)	133 (84.2)
	No	31 (20.7)	25 (15.8)
Trust health care worker on malaria treatment	Yes	126 (78.3)	129 (80.1)
	No	35 (21.7)	32 (19.9)
Alcohol drinking	Yes	43 (26.7)	43 (26.7)
	No	118 (73.3)	118 (73.3)
Member of the women development group	Yes	41 (25.5)	42 (26.1)
	No	120 (74.5)	119 (73.9)
Member of community-based health insurance	Yes	100 (62.1)	99 (61.5)
	No	61 (37.9)	62 (38.5)
Availability of road to the health facility	Yes	100 (62.1)	101 (62.7)
	No	61 (37.9)	60 (37.3)
Time to health facility	< 30 min	71 (44.1)	77 (47.8)
	> 30 min	90 (55.9)	84 (52.2)
Distance to health facility	< 5 km	66 (41)	76 (47.2)
	> 5 km	95 (59)	85 (52.8)
Type of transport	Car	51 (31.7)	58 (36)
	Motor cycle	3 (1.9)	4 (2.5)
	Foot	107 (66.5)	99 (61.5)

### Determinants of malaria treatment delay

Gender, age, residence of place, educational status, medication-taking at home for the current illness, personally making the decision for malaria treatment-seeking, dead family member in the last month due to any cause, and having good or poor knowledge on malaria symptoms, transmission, and malaria at-risk population were found associated with malaria treatment delay on bivariate logistic regression at P-value of ≤ 0.20. In multivariable analysis residence place of the patient, educational status, decision-making on malaria treatment-seeking and knowledge on malaria symptoms were significant predictors of malaria treatment delay at P-value of ≤ 0.05 (Table 3).

**Table 3:** Determinants of malaria treatment delay in the northwestern zone of Tigray, Northern Ethiopia, 2018

Variable	Category	Cases n = 161 No (%)	Control n = 161 No (%)	COR	AOR
Sex	Female	116 (72)	105 (65.2)	1.38 (0.86–2.21)*	0.81 (0.46–1.45)
	Male	45 (28)	56 (34.8)	1.00	1.00
Age	< 7	17 (10.6)	27 (16.8)	1.00	1.00
	7–14	19 (11.8)	21 (13)	1.44 (0.60–3.42)	1.61 (0.52–4.98)
	15–45	110 (68.3)	101 (62.7)	1.73 (0.89–3.36)	0.93 (0.28–3.07)
	> 45	15 (9.3)	12 (7.5)	1.98 (0.75–5.25)	1.1 (0.25–4.81)
Address	Laelay Adyabo	91 (56.5)	90 (55.9)	1.48 (0.84–2.6)*	1.46 (0.73–2.92)
	Tahtay Adyabo	42 (26.1)	30 (18.6)	2.05 (1.05–4.01)*	2.84 (1.29–6.27)**
	Asgede Tsimbla	28 (17.4)	41 (25.5)	1.00	1.00
Educational status	No formal education	85 (52.8)	71 (44.1)	1.73 (0.88–3.41)*	2.39 (1.09–5.22)**
	Primary education	58 (36)	64 (39.8)	1.31 (0.65–2.63)	1.58 (0.74–3.41)
	Above secondary	18 (11.2)	26 (16.1)	1.00	1.00
Medicine taking in home	Yes	10 (6.4)	20 (12.4)	0.47 (0.21–1.03)*	0.43 (0.18–1.02)
	No	151 (93.6)	141 (87.6)	1.00	1.00
Who decide for health care seeking	Patients	82 (50.9)	72 (44.7)	1.85 (0.92–3.72)*	2.38 (1.09–5.2)**
	Father	63 (39.1)	63 (39.1)	1.62 (0.8–3.32)*	2.52 (1.13–5.62)**
	Mother	16 (9.9)	26 (16.1)	1.00	1.00
Dead family member in the last month	Yes	2 (1.2)	6 (3.7)	0.32 (0.06–1.63)*	0.43 (0.08–2.28)
	No	159 (98.8)	155 (96.3)	1.00	1.00
Knowledge on malaria symptoms	Good	74 (46)	55 (34.2)	1.64 (1.05–2.57)*	2.02 (1.21–3.39)**
	Poor	87 (54)	106 (65.8)	1.00	1.00
Knowledge on malaria transmission	Good	142 (88.2)	133 (82.6)	1.57 (0.84–2.95)*	1.15 (0.53–2.48)
	Poor	19 (11.8)	28 (17.4)	1.00	1.00
Knowledge of malaria at risk population	Good	62 (38.5)	82 (50.9)	0.6 (0.39–0.94)*	0.83 (0.45–1.52)
	Poor	99 (61.5)	79 (49.1)	1.00	1.00

* Significant variables at P-value < 0.2

** Significant variable at P-value ≤ 0.05

Malaria patients who resided in Tahtay Adyabo were more likely to delay seeking treatment than those who resided in Asgede Tsimbla district. Regarding educational status, patients who didn’t attend formal education were more likely to delay seeking malaria treatment than those who attended at a level above the secondary level. The odds of malaria treatment delay were more likely higher among the patient who decided to seek health care by themselves and by fathers of less than 18 years old patients than who decided by mothers of less than 18 years old patients. Malaria patients who had good knowledge of malaria symptoms were two times more likely to delay seeking malaria treatment than those who had poor knowledge (Table 3).

## Discussion

The overall result of this study indicated that being a resident of Tahtay Adyabo, educational status, a decision on malaria treatment, and knowledge on malaria signs and symptoms were significant predictors of malaria treatment delay.

Location of residence of a patient was a risk factor for delaying the timing of malaria treatment. Malaria patients residing Tahtay Adyabo were more likely to delay seeking malaria treatment than those who residing in Asgede Tsimbla district. This was supported by the study conducted in west Ethiopia, Uganda and sub Saharan Africa which all found a delay in treating malaria based on geographic location [15, 28, 29]. In this study, Tahtay Adyabo district has more lowland areas making those health facilities difficult to access as clients have to travel long distances on foot.

Educational status of patients seeking care to malaria treatment was a significant factor affecting the timing of seeking treatment of malaria. Patients who had not attended formal education were more likely to be delay seeking malaria treatment than those who attended secondary and above level of education. This result is consistent with the studies conducted in southern Ethiopia, western Ethiopia, and sub Saharan Africa [15, 18, 29]. The relationship between literacy level and early health-seeking may be because people who are literate are better able to access and understand health education messages from different media and health professionals. Although this finding is different from the Nigerian experience [24] it could be that the small sample size in the Nigerian study was unable to reveal this link.

The decision about health-seeking was also found to be affected by the household partner/head. Malaria treatment delay was more likely higher among patients who decided by themselves to seek treatment, and if the decision was being made by the father of a patient < 18, than if the decision was made by mothers of patient < 18. This result is similar to studies conducted in Tanzania and Ethiopia where treatment of malaria was influenced by the head of the household [30, 31]. The reason for this difference may be because women’s work is close to the home they have more chances to identify symptoms early and economic condition of people (farmers and students) that they delay seeking care for themselves.

When examining the impact of knowledge malaria upon treatment-seeking, our study malaria found that patients who have good knowledge of malaria symptoms were two times more likely to delay seeking malaria treatment than those who had poor levels of knowledge on malaria symptoms. Although this result is similar to a previous study in Ethiopia [32], it is contrary to that found in studies conducted in eastern Rwanda and northwest Ethiopia [26, 33]. This difference may be related to the distance. In this study, most of the participants with good knowledge of malaria symptoms come from greater than 5 km distance for the health facility, and an area with difficult geographical terrain, both which cause difficulties for them to seek timely treatment. But this could also be a factor in delaying care-seeking by those participants who had poor knowledge of malaria. Further studies should investigate this in more detail to better define the corrective strategies required.

A limitation of this study could be recall bias regarding the timing of onset of symptom. The study attempts to minimize this bias by using the socially defined time period to better estimate the duration of the period in past events. There may have also been interviewer bias due to interviewer familiarity with the cases and controls. The study sought to minimize this type of error through the detailed training provided to the interviewers and the supervision and cross-checking used in the study during data collection.

## Conclusion

The study found a strong linkage between delay in seeking care and location of residence of patients, educational status, the person who decided to seek treatment of the patient, and level of knowledge of malaria symptoms. Therefore better to improve access to health facilities for early treatment of malaria in these locations where the community must walk long distances to seek health care. Additionally better understanding of the effectiveness of the existing awareness programs on malaria, its symptoms and treatment, and the need for early diagnosis and treatment, acceptability and empowerment of the service is crucial. There appears to be diversity in levels of understanding of these issues amongst the study population, therefore needs delivery of messages in different way among different literacy levels.

## Data Availability

Data are available from the corresponding author on reasonable requests.

## Abbreviations

AOR: adjusted odds ratio
COR: crude odds ratio
CI: confidence interval
FMOH: Federal Ministry Of Health
PMI: President Malaria Initiative
RDT: rapid diagnostic test
VIF: variance inflation factor
WHO: World Health Organization
